# Assessment of knowledge and awareness of safe disposal of unused or expired medication in Saudi Arabia: A cross-sectional study

**DOI:** 10.1016/j.jsps.2022.09.012

**Published:** 2022-09-30

**Authors:** Abdulhamid Althagafi, Mohannad Alshibani, Samah Alshehri, Ahmed Noor, Alaa Baglagel, Tahani Almeleebia

**Affiliations:** a Faculty of Pharmacy, Department of Pharmacy Practice, King Abdulaziz University, Jeddah, Saudi Arabia; b College of Pharmacy, University of Arizona, Tucson, United States; c Faculty of Pharmacy, Department of Clinical Pharmacy, King Khalid University, Abha, Saudi Arabia

**Keywords:** Medication disposal methods, Pharmaceutical waste, Expiry medicines, Environmental hazard

## Abstract

**Background:**

Poor waste management of unused or expired medications jeopardizes healthcare staff, employees who oversee medical waste, patients and their families, the neighboring population, and environmental contamination. In addition, the inappropriate treatment or disposal of that waste leads to. In addition, medical waste disposal exerts an intolerable burden on the economy of health care facilities. Currently, there is a lack of data in community settings regarding adequate methods of medication disposal in Saudi Arabia.

**Aim of the Study:**

The current study aimed to evaluate current knowledge and awareness of the safe disposal of unused or expired medicines in the Saudi Arabia.

**Method:**

A survey study was conducted in Saudi Arabia within 5 months from October 2021–February 2022. The survey was distributed to participants via social media channels. The questionnaire was constituted of 16 items divided into three sections: demographic information, quantification, and characterization of unused and expired medication at home, and practice and attitude regarding the disposal of unused or expired medication.

**Results:**

The survey was taken by 1105 participants and 1100 (99.54%) participants completed the survey. The study found that (49.1%) of participants stored medicines at home and these medicines were mainly stored in the refrigerator (64.4%). Household trash was the most frequent method of disposal (79.5%). Non-prescribed medicines (67%) were mainly stored as unused or expiry medicines at home followed by prescribed medicines (51.9%). The main reason for the storage of unused/expired medicines at home was stopped medication after recovery (68.5%). Only 8.4% of participants had received appropriate education or training related to the correct disposal of medication. The best-practiced method to increase community awareness regarding the disposal of unused or expiry medicine was awareness through social networking (70.3%). In conclusion, patients’ education regarding safe medication disposal and availability of medication disposal program is necessary to improve appropriate medication waste methods and decrease possible environmental harm.

## 1 Introduction

Current healthcare systems are directed at providing patients with the best healthcare quality. In the way to achieve this goal, maximum utilization of the available resources and reduction of monetary losses are attained through disease prevention, managing patients, and saving lives (Braund et al., 2008). Medical waste disposal includes unused medications such as contaminated, spilled, or expired drugs, vaccines, or other pharmaceutical products. The disposed waste carries a significant risk to the individual health status through acquiring infection, trauma, and exposure to chemicals and radiation. Furthermore, it should be highlighted that poor medical waste disposal impacts healthcare staff, employees overseeing the disposed of materials, and patients as well as results in environmental contaminations endangering the health of humans, animals, and plants (Insani et al., 2020).

The waste disposal of hospital and health facilities are composed of refuse similar to household waste (75–90%) and hazardous medical waste (10–25%) (Chartier, 2014). Hazardous medical waste can be classified into 5 categories: (1) sharp objects carrying risk of injury; (2) contaminating waste such as blood, excreta, and body parts; (3) pharmaceutical waste entailing expired, spilled, and unused medications as well as batteries, mercury waste, and laboratory materials; (4) pressurized containers such as gas cylinders and aerosol cans; (5) radioactive waste (World Congress on Engineering and Computer Science (WCECS), 2013). It is noteworthy to emphasize the global challenge developed due to the lack of authorized regulations, no proper education provided to the patients concerning the medications disposal, and the erroneous understanding of the term “expired” labeling the medications packs (Alnahas et al., 2020).

For the Arabian Gulf countries, including Saudi Arabia, medical waste adds an unbearable burden on the cost of healthcare while sustaining the same high quality of healthcare services (Chew et al., 2021). Nearly 5% of the IV admixtures prepared and distributed in intensive care units were expected to be discarded (Alrashed et al., 2021). In Saudi Arabia, the medications are preferably discarded in the home garbage (75.75%), sewage system, or kept until expiration (Abdullah, 2018). Furthermore, the majority of the Saudi population needs to be provided with adequate knowledge on how to properly dispose of unwanted or expired medications. It was found that only 3% of expired medications were returned to the dispensing medical facility and most of the Saudi population disposes of the medical waste through household trash (Abuassonon et al., 2019).

Therefore, an increasing number of community-based organization adopts the “take-back” program as a favorable option for waste medical disposal. Disposal of waste medicines in household trash is only accepted when certain precautions are considered. Nevertheless, the policy of “take-back” medicines is not applied in Saudi Arabia (Jafarzadeh et al., 2021, Kelly et al., 2018). Therefore, it is recommended that providing the proper advice to the population and the development of an organized method for collecting the discarded medications would be beneficial (Banjar et al., 2022). In this regard, most of the Saudi population (90.66%) welcomed the implementation of the “take-back” strategy as an approach to safely discard unused or expired medications (Abuassonon et al., 2019).

The purpose of the current study was to assess knowledge and awareness of the safe disposal of unused or expired medications in Saudi Arabian community.

## 2 Methods

This survey study was conducted over 5 months from October 2021-February 2022 in Saudi Arabia. The subject eligible to take the survey were adult participants more than 18 years old and residents of Saudi Arabia. Exclusion criteria were participants aged less than 18 years of age or refusing to participate in the survey. The goal of the study was explained to all participating individuals. A link to the electronic survey was sent to participants via social media that directed them to a Google form in which participants completed the survey. Before starting to answer survey questions, participants were asked to consent to participate in the survey. The questionnaire took an average of 10 min to complete.

The original questionnaires were distributed in Arabic language and then translated into English for publication. The questionnaire contained 16 items divided into three sections: Section 1: demographic information; Section 2: quantification and characterization of unused and expired medication at home; and Section 3: practice and attitude regarding the disposal of unused or expired medication. Most of these questions were assessed utilizing a 5-point Likert scale of 1 to 5. Questions 6–7, 9–10 involved the terms: 1 = always, 2 = often, 3 = sometimes, 4- rare, and 5 = never. Questions 13–14, 16–18 involved the terms: 1 = highly agree, 2 = agree, 3 = don’t agree, 4 = disagree, 5 = strongly disagree. Question 21 involved the terms: 1 = yes, 2 = no, and 3 = don’t know. Since this survey was an observational cross-sectional study, therefore the results were reported in numbers and percentages using Microsoft excel.

## 3 Results

The survey was distributed to 1105 participants and all participants has responded to the survey (100% response rate). The demographic characteristics are demonstrated in Table 1.

**Table 1:** Demographic characteristics.

PARAMETER	N = 1105 (%)
Gender	
• Male	428 (38.9%)
• Female	672 (61.1%)

Age	
• Less than 18	5 (0.5%)
• From 19 to 29	273 (24.8%)
• From 30 to 39	325 (29.5%)
• From 40 to 49	237 (21.5%)
• Above 50	260 (23.6%)

The highest educational level	
• Less than high school	66 (6%)
• High school	284 (25.8%)
• Diploma	128 (11.6%)
• Bachelor	546 (49.6%)
• Master	57 (5.2%)
• PhD	19 (1.7%)

Social status	
• Married	662 (60.2%)
• Single	438 (39.8%)

Resident of any region	
• Makkah	874 (79.5%)
• Riyadh	101 (9.2%)
• Eastern region	31 (2.8%)
• Qassim	17 (1.5%)
• Medina	30 (2.7%)
• Asir	12 (1.1%)
• Tabuk	6 (0.5%)
• Jizan	14 (1.3%)
• Baha	5 (0.5%)
• Jouf	4 (0.4%)
• Hail	3 (0.3%)
• Northern borders	2 (0.2%)
• Najran	1 (0.1%)

Frequency of medications storage at household	
• Always	540 (49.1%)
• Often	259 (23.5%)
• Sometimes	215 (19.5%)
• Rare	62 (5.6%)
• Never	24 (2.2%)

Storage location at household	
• Locker	216 (19.6%)
• Refrigerator	708 (64.4%)
• Bedroom	441 (40.1%)
• Living room	53 (4.8%)
• I do not know	8 (0.7%)
• Medicine cabinet	281 (25.5%)

If needed, storing of medications away from moisture, light, and high temperature (more than 25 °C) is practiced in household	
• Always	490 (44.5%)
• Often	286 (26%)
• Sometimes	182 (16.5%)
• Rare	88 (8%)
• Never	54 (4.9%)

Number of unused and/or expired medications stored in household	
• 0–5	787 (71.5%)
• 6–10	196 (17.8%)
• 11–15	43 (3.9%)
• 16–20	15 (1.4%)
• More than 20	59 (5.4%)

Classes of expired and unused medications that are in your household	
• Prescribed medicines	571 (51.9%)
• Non-prescribed medicines	737 (67%)
• Subcutaneous injection	49 (4.5%)
• Intramuscular injection	37 (3.4%)
• Intravenous	18 (1.6%)
• Patches	146 (13.3%)
• Others	214 (19.5%)

Reasons for keeping unused/expired medicines at home (choose all applicable answers)	
• The medicine has been changed by the doctor	394 (35.8%)
• The amount of medication dispensed is more than needed	474 (43.1%)
• Stop using medication after recovery	754 (68.5%)
• The prescribed medication has unpleasant side effects and has been discontinued	257 (23.4%)
• The medication has expired	131 (11.9%)
• Other reasons	130 (11.8%)

Use of any prescribed medication for a relative or a friend	
• Always	34 (3.1%)
• Often	74 (6.7%)
• Sometimes	354 (32.2%)
• Rare	288 (26.2%)
• never	350 (31.8%)

Verification of expiry date before administering medications	
• Always	773 (70.3%)
• Often	130 (11.8%)
• Sometimes	105 (9.5%)
• Rare	56 (5.1%)
• Never	36 (3.3%)

Participants who tend to store medications at home were 92.1%. Participants who tend not to store medications at home (responding by rare and never) were 7.8% (Table 1). The refrigerator was the most storage place at home for medication chosen by 64.4% of the participants followed by the bedroom, 40.1%. Only 0.7% of the participants responded with “I do not know” (Table 2). Keeping medicine away from moisture, light, and high temperature (>25 °C) was reported by an overall 87% of the participants who tend to choose the proper way of medication storage. An overall 12.9% of the participants do not store medications properly (Table 2).

**Table 2:** Awareness and knowledge of medication disposal.

PARAMETER	N (%)
Current medications disposal methods	
• household trash bins	874 (79.5%)
• Toilet flush	38 (3.5%)
• Return to pharmacy	44 (4%)
• Return to hospital	42 (3.8%)
• Give/donate medications to other people	142 (12.9%)
• I do not dispose medications	75 (6.8%)
• Other methods	45 (4.1%)

Received education regarding safe disposal of medications?	
• Yes	92 (8.4%)
• No	960 (87.3)
• Don’t know	48 (4.4%)
• Strongly disagree	6 (0.5%)

Seeking information from official resources regarding safe disposal of medications	
• Always	139 (12.6%)
• Often	99 (9%)
• Sometimes	213 (19.4%)
• Rare	202 (18.4%)
• Never	447 (40.6%)

Responsible sector to educate the public about safe disposal of medications	
• Hospitals	525 (47.7%)
• Pharmacies	587 (53.4%)
• Social media	449 (40.8%)
• Medical waste companies	547 (49.7%)

Unsafe disposal of medications associated with harmful consequences on public health and global environment	
• Highly agree	562 (51.1%)
• Agree	315 (28.6%)
• Don’t know	204 (18.5%)
• Disagree	13 (1.2%)
• Strongly disagree	0 (0%)

The presence of designated medication disposal locations or medications disposal process will improve safe medication waste management practices	
• Highly agree	750 (68.2%)
• Agree	285 (25.9%)
• Don’t know	51 (4.6%)
• Disagree	12 (1.1%)
• Strongly disagree	2 (0.2%)

Locating medications disposal facilities via smart phone application would assist in safe drug disposal practice	
• Highly agree	751 (68.3%)
• Agree	277 (25.2%)
• Don’t know	50 (4.5%)
• Disagree	19 (1.7%)
• Strongly disagree	3 (0.3%)

Effective methods to raise the community awareness regarding medications disposal	
• Deliver clear instructions while patients receive medications	758 (68.9%)
• Patient education by health care professionals	488 (44.4%)
• Public awareness via social media	773 (70.3%)
• Other methods	110 (10%)

Best method to dispose medication in from participants point of view	
• Return to pharmacy	250 (22.7%)
• Take it to the hospital	181 (16.5%)
• Through household trash	457 (41.5%)
• Through toilet flush	43 (3.9%)
• Others	169 (15.4%)

Participants’ opinion about disposing unused/expired medications?	
• Highly agree	872 (79.3%)
• Agree	165 (15%)
• Don’t know	37 (3.4%)
• Disagree	20 (1.8%)
• Strongly disagree	6 (0.5%)

Participants’ opinion on if the presence of smartphone applications locating drug disposal facilities would assist in safe medication disposal practice	
• Highly agree	751 (68.3%)
• Agree	277 (25.2%)
• Don’t know	50 (4.5%)
• Disagree	19 (1.7%)
• Strongly disagree	3 (0.3%)

Participants’ opinion on the best method to raise community awareness regarding medication disposal	
• Deliver clear instructions while the patient receives the drug	758 (68.9%)
• Patients’ education by a health care practitioner	488 (44.4%)
• Publicize awareness through social network	773 (70.3%)
• Other ways	110 (10%)

Our study revealed that most participants disposed of unused and expired medications via household droppings 79.5%, followed by donating the unused medications to others 12.9%. On the contrary, 6.8% of participants did not dispose the unused/expired medications (Table 2).

Only 8.4% of participants had received appropriate education or training related to the correct disposal of medication. It was agreed by 70.3% of participants that the best way to raise community awareness regarding medication disposal should be through social media. Others preferred to deliver clear instructions while receiving the medications or from healthcare practitioners; 68.9% and 44.4% respectively. Most of the participants 59% did not seek information regarding the safe disposal of medications. Moreover, participants believe that pharmacies (53.4%), followed by hospitals (47.7%) are responsible for public education about the safe disposal of medications (Table 2).

Concerning the belief in the harmful consequences of unsafe disposal of medical waste on public health and the global environment, an overall 79.7% of participants tended to believe in the harmful consequences of unsafe disposal of medical waste on public health and global environment. Additionally, 94.1% of respondents supported the presence of a mechanism for medication waste disposal. The smartphone application was believed that it would assist in safe drug disposal practice by locating drug disposal facilities by 93.5% of participants and 6.5% of participants were against the idea (Table 2).

For the awareness of the best way to dispose of medical waste, most participants (42.5%) reported disposal through household trash, followed by reported returning to the pharmacy as the best way for waste disposal (22.7%), (16.5%) preferred to take discarded medicine to the dispensing hospital, and only (3.9%) reported disposal through toilet flush as the best method for medical waste disposal (Table 2).

## 4 Discussion

The WHO guidelines for safe storage practice of medicine emphasize keeping medications in a clean, dry store and within recommended temperature limits and away from the children's reach (UNICEF and World Health Organization (WHO, 2003)). The refrigerator, medical cabinet, and First Aid box were the topmost places reported for storage of medicine (Jafarzadeh et al., 2021). The current study reflects relatively high awareness of participants about the safe and proper storage practice of medications as the refrigerator was the topmost storage place at home for medications (64.4%). Moreover, the current study reflects the awareness of participants of the expiry date as nearly 91% of participants checks the expiry date before taking the medications.

It was reported that the prevalence of medication storage at home ranged from 35% up to 90% (Garg and Karan, 2009, Jafarzadeh et al., 2021). Tablets, syrup, suspension, and capsule drugs were the topmost medicine forms that tend to be stored at home (Mirza and Ganguly, 2016). The current study showed no exception; 92.1% of the participants tend to store medications at home. Most of the stored medicine is prescribed and non-prescribed medicine (tablets, syrup, capsules) while injections constitute the minority of stored drugs.

Inappropriate use and/or disposal of medical waste is of public health concern (Kelly et al., 2018). A large body of literature provided strong evidence that 97% of expired (and unused or leftover) medicines were kept at home for future use by the households irrespective of the global income of the country (Dayom et al., 2014, Paut Kusturica et al., 2016). Contrary to the literature, awareness of the expiry date is high as an overall of the participants (91.6%) do check the expiry date before taking the medications.

Household trash was reported to be the most common method for disposing of discarded (unused or expired) medications worldwide both in developed and developing countries with a prevalence ranging from 3% in undeveloped countries to nearly 100% in developing countries (Wang et al., 2021). A recent study concurred with the preference of household trash for medication disposal (26.27%) while returning medications to the pharmacy (12%) is hardly considered (El-hamamsy, 2011). Flushing down the drain is ranked the second leading method for medical waste disposal, especially for the liquid medication (28%) (Fenech et al., 2013, Labu et al., 2013, Yu et al., 2019). In some countries such as Sweden and Germany, discarded medications were reported to return to the pharmacy for proper disposal (Goetz and Keil, 2007, Persson et al., 2009).

The current study agreed with the worldwide percentage as the household droppings were reported by 79.5% of the participants to be the preferred way of discarding medications disposal and only 3.5% of the participants believed that flushing the toilet is the best way for medications disposal. Contrary to the Sweden and Germany studies the current study found that the Saudi population (7.8%) is less likely to return the discarded medication to the pharmacy or hospital (Kinrys et al., 2018, Paut Kusturica et al., 2017). Moreover, it was found that a small percentage (12.9%) of participants tend to pass or donate unused medication to others. However, the Saudi population (39.2%) are not lacking awareness of the need for safe disposal by returning the discarded medicine to the pharmacy or the dispensing hospital. Most of the respondent (94.3%) agreed with the need for safe medication disposal. However, there is no functioning take-back program to collect unused and expired medications to be incinerated safely (Kusturica et al., 2012, Vellinga et al., 2014).

Although the awareness of environmental safety influences the choice of safe means for medication waste disposal, the behavior of the population is far from equating to the level of awareness and information received (Paut Kusturica et al., 2017). The perception of the individual of the impact of one’s action on the environmental safety and public health takes part in the individual behavior including safe medical waste disposal (Gifford and Nilsson, 2014). Studies found that there is a discrepancy between the information on the proper medical waste disposal and the disposal behavior (Kotchen et al., 2009, Persson et al., 2009). In the current study, most participants (41%) tend to seek information about safe medical waste disposal from official resources including hospital, pharmacies, and medical waste companies. This information-seeking behavior reflects that the Saudi population (79.7%) is knowledgeable about the harmful consequences of unsafe disposal of medication waste on public health and the global environment. Moreover, most of the population supports the presence of a mechanism for medication waste disposal including a smartphone application. However, their practice is not equating the good intentions as only the minority return the discarded medicine to pharmacies or hospitals, less than 9% of the participants.

Improper disposal of medication waste leads to environmental pollution with pharmaceuticals. Surface water and underground aquifers were found to be contaminated with pharmaceuticals that would find access to the living beings (Kinrys et al., 2018, Kinrys et al., 2016). In Saudi Arabia, the majority of the population (70.20%) actively sought a safe and appropriate method for medical waste disposal with an interest (78%) in receiving the right information for the proper disposal of medical waste (Al-Shareef et al., 2016). In a recent study, 75% of participants admitted the lack of enough information related to the proper disposal of medical waste and concurred on the need for further awareness and education (Gidey et al., 2020).

In accordance with the literature here before, the current study showed that almost all the participants (87.3%) received no education related to appropriate methods to dispose of unused/expired medications. However, participants suggested several community-based ways to raise population awareness about medical waste disposal appropriately including social networking (70.3%), delivering clear instructions while the patient receives the drug (68.9%), and patient education by a health care practitioner (44.4%).

The main strong point of the current study is the updated characterization of the attitude of the Saudi population towards medical waste disposal… The main limitation is the potential bias inherited in the survey studies in general. In additions, even though participants were from most of the county’s provinces and area; however, most of them were form Makkah and western regions. Consequently, it is difficulty to assume that the result of our study reflects most of Saudi population.

The Saudi population presented in the participants is relatively aware of the problem of medical waste storage and disposal. The provision instructions concerning the proper storage and disposal should be intensively provided through several means including social medical and digital applications. Moreover, pharmacies and hospitals should adopt a take-back program for increasing the awareness and safe disposal of medical waste. Further studies are needed to confirm the results.

## 5 Conclusion

In Conclusion, the study revealed that implementing effective patients’ education programs regarding appropriate methods of medication disposal targeting the community along with the presence of effective and readily available medication wastage program play a crucial part in safe medications disposal and preventing environmental hazardous waste exposure. In addition, the majority of our participants have the necessary knowledge about safe medication storage.

## Funding

This research work was funded by Makkah Digital Gate Initiative under Grant No. (MDP-IRI-4-2020). Therefore, the authors gratefully acknowledge technical and financial support from the Emirate Of Makkah Province and King Abdulaziz University, Jeddah, Saudi Arabia.

## Declaration of Competing Interest

The authors declare that they have no known competing financial interests or personal relationships that could have appeared to influence the work reported in this paper.
